# Transport and Response Coefficients in Second-Order Dissipative Relativistic Hydrodynamics with Quantum Corrections: Probing the Quark–Gluon Plasma

**DOI:** 10.3390/e27060580

**Published:** 2025-05-29

**Authors:** Iberê Kuntz, Roldao da Rocha

**Affiliations:** 1 Departamento de Física, Universidade Federal do Paraná, P.O. Box 19044, Curitiba 81531-980, PR, Brazil; kuntz@fisica.ufpr.br; 2 Center of Mathematics, Federal University of ABC, Santo André 09210-580, SP, Brazil

**Keywords:** hydrodynamics, quark–gluon plasma, transport and response coefficients, nonequilibrium quantum field theory

## Abstract

A functional measure encompasses quantum corrections and is explored in the fluid/gravity correspondence. Corrections to the response and transport coefficients in the second-order dissipative relativistic hydrodynamics are proposed, including those to the pressure, relaxation time, and shear relaxation time. Their dependence on the quark–gluon plasma (QGP) temperature sets a temperature dependence on the running parameter encoding the one-loop quantum gravity correction, driven by a functional measure. The experimental range of the bulk-viscosity-to-entropy-density ratio of the QGP, obtained by five different analyses (JETSCAPE Bayesian model, Duke, Jyväskylä–Helsinki–Munich, MIT–Utrecht–Genève, and Shanghai) corroborates the existence of the functional measure. Our results suggest that high-temperature plasmas could be used to experimentally test quantum gravity.

## 1. Introduction

AdS/CFT duality relates strongly correlated quantum field theories with weakly coupled classical gravity. In the original setup, AdS/CFT states a well-defined relation between a four-dimensional conformal field theory (CFT) and the geometry of a five-dimensional anti-de Sitter (AdS) background describing gravity [1,2,3]. Since the classical dynamics of weakly coupled gravity are described in a codimension-one spacetime, AdS/CFT is inherently a holographic duality and the formal inception of other relevant gauge/gravity correspondences, including AdS/QCD and AdS/CMT.

When collective phenomena are studied in QCD and condensed matter, one can observe that strongly coupled quantum systems rearrange themselves in such a way that weakly coupled degrees of freedom dynamically emerge. Hence, a quantum system can be better formulated with respect to the fields corresponding to the new emergent excitations. Gauge/gravity holographic dualities engender straightforward examples of this pattern, where the regarded emergent fields regulate gravity living in a codimension-one spacetime. The additional dimension can be thought of as the energy scale of the dual quantum field theory. Gauge/gravity dualities encompass a variety of quantum systems, including, for instance, the strongly coupled dynamics in QCD, black-hole physics, quantum gravity, relativistic hydrodynamics implementing the fluid/gravity correspondence, and holographic superconductors in condensed matter physics, among others [4].

AdS/CFT can be analyzed in the long-wavelength limit, implementing a special type of gauge/gravity duality, known as the fluid/gravity correspondence. In this context, finite-temperature quantum systems, such as the quark–gluon plasma (QGP), can be studied using relativistic hydrodynamics. In this setup, the main features of viscous fluid flows can be specified by their transport and response coefficients, which enclose the relevant microscopic features of the relativistic hydrodynamics controlling the fluid flows. Dual to the hydrodynamics on the boundary, gravity in the codimension-one bulk can be characterized by an AdS black brane containing (at least) one non-degenerate event horizon. Therefore, the conformal field theory on the boundary, in the long-wavelength limit, is governed by the near-horizon regime of gravity in the bulk geometry [5]. AdS/CFT is thus a very advantageous instrument for calculating the transport and response coefficients in relativistic hydrodynamics [6].

Fluid/gravity correspondence establishes that the energy-momentum tensor describing the strongly coupled boundary CFT is dual to the operators representing graviton fields in the AdS bulk. In the long-wavelength limit, the fact that the energy-momentum tensor is conserved leads to the relativistic hydrodynamical description of fluid flows. In this way, Einstein’s equations in the AdS bulk can be related to the Navier–Stokes equations on the boundary [7,8,9,10,11,12,13,14]. Several properties and features of relativistic hydrodynamics on the AdS boundary, describing a viscous fluid flow characterized by its response and transport coefficients, have been scrutinized [15], also comprising soft-hair excitations [16,17] and fermionic sectors [18,19]. Relativistic hydrodynamics is also a useful tool for studying phase transitions and deconfinement in QCD [20,21,22,23,24]. Ref. [25] regarded black-hole solutions with equations of state resembling those in QCD at zero chemical potential, and the shear-viscosity-to-entropy-density and bulk-viscosity-to-entropy-density ratios were calculated. Refs. [26,27] studied anisotropic shear viscosity in strongly coupled plasmas with an external magnetic field, whereas momentum transport was scrutinized in this context in Ref. [28]. Ref. [29] investigated the QGP from the point of view of the Einstein–Born–Infeld dilaton model, whereas other aspects of holography were investigated in this context [30,31,32,33]. Ref. [34] scrutinized the Bayesian analysis of the QGP bulk and shear viscosities at the high-chemical-potential region.

The use of relativistic viscous hydrodynamics to model the evolution of the QGP generated in ultrarelativistic heavy-ion collisions is currently ubiquitous. Important results using computational simulations comply with experimental data available from the Relativistic Heavy-Ion Collider (RHIC) [35]. One of the most straightforward yet successful models describing heavy-ion collisions is the liquid-drop model, whose evolution is governed by the equations of motion in relativistic hydrodynamics. For them to be applied, the characteristic length scale of the system under scrutiny must, in general, be much larger than the mean free path ($\ell_{\mathrm{MFP}}$). In this way, relativistic hydrodynamics consists of an effective theory capturing the low-frequency dynamics of wave modes with frequency and momentum much smaller than the inverse of $\ell_{\mathrm{MFP}}$. Nuclear physics described by QCD has the mean free path of Fermi order, and relativistic viscous hydrodynamics works reasonably well to study collective phenomena in strongly interacting matter produced by the RHIC and the LHC [36].

The transport and response coefficients in fluid dynamics measure how fast a perturbed system returns to equilibrium and are intrinsic tools underlying the hydrodynamical description. In the non-relativistic Navier–Stokes formulation, the dissipative currents, encompassing the heat flow, bulk viscous pressure, and shear energy-momentum tensor, are supposed to have a linear dependence on the forces. They are indeed represented by the gradients of the fluid 4-velocity, temperature, and chemical potential. The associated parameters of proportionality are the bulk viscosity, shear viscosity, and heat conductivity. The Navier–Stokes description can be extended by considering higher-order gradients of the 4-velocity, temperature, and chemical potential, leading to the bulk and shear relaxation times, setting the characteristic time scales for dissipative currents to relax to their respective first-order solutions. The field-theoretical origin of the shear relaxation time was reported in Ref. [37], which showed the microscopic emergence of the shear relaxation time. Kubo’s formula for the shear relaxation time was obtained for systems with conformal symmetry in Refs. [38,39,40], calculating the response of the referred systems to small perturbations for a background metric. Also, Kubo’s formula for the product of the bulk viscosity and the bulk relaxation time can be deduced from the response functions studied in Ref. [41].

Ref. [42] also studied quantum gravity corrections due to the functional measure to some transport and response coefficients of the gauge theory. The functional measure is indeed required for the invariance of the effective action under field reparameterizations (and hence under gauge transformations) [43,44,45,46,47,48]. Geometrically, it can be introduced via the determinant of the configuration-space metric, following the same steps to obtain the integration measure in curved spacetimes. Unlike the gravitational analogy, however, the configuration-space metric is fixed from the onset. Thus, the classical action and the configuration-space metric must be given to fully specify a theory, both of which can be determined using effective field theory (One could promote the configuration-space metric to a dynamical object, but it brings back parameterization-dependence problems.). The functional measure so obtained gives a correction to the effective action at one-loop order. In this paper, we build on the work of Ref. [42] by computing the corrections due to the functional measure to other response and transport coefficients.

In the previous analysis in Ref. [42], the calculation of the functional measure’s running parameter γ was based on the comparison of the bulk-viscosity-to-shear-entropy ratio with experimental data of the QGP, including the JETSCAPE Bayesian model [49,50], the analysis by the Duke group [51], and the Jyväskylä–Helsinki–Munich group [52]. In this case, experimental lower and upper bounds on γ were found [42]. In this paper, these analyses are updated and added to the analysis of the bulk-viscosity-to-entropy density of the QGP by the MIT–Utrecht-Genève group [53,54] and the Shanghai group [55]. Moreover, the analysis of other transport and response coefficients can propose to test quantum gravity in high-temperature scenarios, showing that the term due to the functional measure dominates as the temperature increases. A strong result in our current work makes the experimental range of the bulk-viscosity-to-entropy density of the QGP, obtained by five different phenomenological analyses (JETSCAPE Bayesian model, Duke, Jyväskylä–Helsinki–Munich, MIT–Utrecht–Genève, and Shanghai), corroborate the existence of a non-vanishing renormalized parameter encoding the one-loop functional-measure quantum gravity correction.

This paper is organized as follows. In Section 2, we review the construction of the functional measure in AdS/CFT using effective field theory. In particular, we show how it leads to a correction to the classical action. The phenomenology of this correction on the gauge side of the AdS/CFT correspondence is then studied in Section 3 by describing the energy-momentum tensor as a perfect and viscous fluid. In Section 4, we perform the second-order hydrodynamics expansion to obtain further quantum gravity corrections to the transport and response coefficients. We compare these results against numerical data from lattice simulations to constrain the functional-measure correction. In particular, the running parameter that regulates quantum gravity corrections due to the functional measure is obtained as a function of the plasma temperature by analyzing the pressure, relaxation time, and shear relaxation time in lattice QCD underlying the QGP. Section 5 shows how to constrain the parameter responsible for carrying quantum gravity effects from experimental data of the QGP and heavy-ion collisions at the LHC and RHIC. For this, the JETSCAPE Bayesian model will be used [49,50], together with the results by the Duke group [51], the Jyväskylä–Helsinki–Munich group [52], the MIT–Utrecht–Genève group [53,54], and the Shanghai group [55]. These analyses are thoroughly implemented in the context of a functional measure to bind the parameter that is responsible for quantum gravity effects. We then draw our conclusions in Section 6.

## 2. Functional Measure in AdS/CFT

The gauge/gravity correspondence can be succinctly stated via the GKP–Witten relation [2,3]:

(1) $$Z_{\mathrm{GAUGE}}=Z_{\mathrm{GRAVITY}}\;.$$

Indeed, all quantities of interest in quantum field theory can be directly obtained from the generating functional (We adopt DeWitt’s notation, where small-letter indices i=(I,x) include both discrete indices *I*, denoted by capital letters, and the spacetime dependence *x*. Repeated small-letter indices amount to summations over discrete indices and spacetime integrations.):

(2) $$Z\left[J\right]=\int d\mu \left[\varphi \right]e^{i\left(S\left[\varphi^{i}\right]+J_{i}\varphi^{i}\right)},$$

where $S[\varphi^{i}]$ denotes the classical action for the underlying theory and $\varphi^{i}=\varphi^{I}\left(x\right)$ denotes arbitrary fields. Equation (1) thus guarantees the equivalence of the corresponding theories.

As evident from Equation (2), such a duality strongly depends on the definition of the path integral, which, however, lacks a full-fledged mathematical construction, particularly regarding the integration measure. From a geometrical point of view, a general configuration space requires the following definition of the integration measure:

(3) $$d\mu \left[\varphi \right]=D\varphi^{i}\sqrt{\mathrm{Det}\;G_{ij}}\;,$$

where $D\varphi^{i}=\prod_{i}d\varphi^{i}$ and $\mathrm{Det}\;G_{ij}$ denotes the functional determinant of the ultralocal configuration-space metric:

(4) $$G_{ij}=G_{IJ}\left(\varphi \right)\;\delta \left(x,x^{\prime }\right)\;.$$

Ultralocality, namely the fact that $G_{ij}$ is proportional to the Dirac delta, is required by consistency with the local *S*-matrix theory. The functional determinant in this case evaluates to

(5) $$\ln\mathrm{Det}\;G_{ij}=\delta^{\left(n\right)}\left(0\right)\int d^{n}x\sqrt{-g}\mathrm{tr}\ln G_{IJ}\;.$$

The term $\sqrt{\mathrm{Det}\;G_{ij}}$ is usually omitted in the path integral when dimensional regularization is used, in which case one writes $\delta^{\left(n\right)}\left(0\right)=0$. However, the situation is not straightforward for such extreme divergences. Dimensional regularization hides the problem behind formal manipulations, not allowing a careful analysis of the path integral measure. It might not even be applicable in these cases.

In the Wilsonian effective field theory, one regularizes the Dirac delta by implementing a cutoff. This can be done, for example, by using a Gaussian distribution, such that

(6) $$\delta^{\left(n\right)}\left(0\right)=\frac{\Lambda^{n}}{{\left(2\pi \right)}^{n/2}}\;.$$

With this regularization, Equation (5) yields a non-trivial contribution from the configuration-space metric in (3) to the effective action. Its contributions to some transport coefficients have already been calculated.

After introducing the non-trivial functional measure, one immediately faces the problem of determining the configuration-space metric. We stress that the choice of this metric must be seen as part of the definition of the theory, along with the bare Lagrangian. Nonetheless, the Wilsonian approach again comes to our rescue. To leading order in the energy expansion, the most general configuration-space metric that satisfies all the required symmetries reads [56]

(7) $$\mathrm{Det}\;G_{ij}∼\prod_{x}\det g_{\mu \nu }\;,$$

whose precise coefficient depends on the fields present in the theory. From Equations (2) and (7), one then finds the contribution of the functional measure to the bare action:

(8) $$Z\left[J\right]=\int D\varphi^{i}e^{i\left(S_{\mathrm{EFF}}\left[\varphi^{i}\right]+J_{i}\varphi^{i}\right)},$$

where (we used the freedom of choosing the normalizing factor Z[0] to include the absolute value in the argument of the logarithm).

(9) $$S_{\mathrm{EFF}}=\int d^{n}x\sqrt{-g}\left(L-i\gamma \;\mathrm{tr}\ln|g_{\mu \nu }|\right)\;,$$

for some bare Lagrangian L. Here, γ is the renormalized parameter, whose Wilsonian renormalization group equation reads

(10) $$\Lambda \frac{dZ}{d\Lambda }=0\;.$$

One should note that the functional-measure correction in Equation (9) is of one-loop order. The usual one-loop correction $\ln\mathrm{Det}\;(S_{,ij})$ combines with the functional-measure correction to wit

(11) $$\ln\mathrm{Det}\;\left(S_{\;,j}^{i}\right)=\ln\mathrm{Det}\;\left(G^{ik}S_{,kj}\right)\;,$$

thus transforming the bilinear term $S_{,ij}$ into the linear operator $S_{\;,j}^{i}$. Only the latter transforms covariantly under field redefinitions, which includes, in particular, spacetime diffeomorphisms. This stresses the fact that, despite the form of the correction in Equation (9), there is no fundamental violation of diffeomorphism invariance. In fact, the functional measure (3) transforms as a functional scalar density, canceling out the functional Jacobian from the transformation of $D\varphi^{i}$. The net transformation thus leaves the path integral invariant under diffeomorphisms.

Because the usual $\ln\mathrm{Det}\;(S_{,ij})$ involves powers of curvatures and/or covariant derivatives, it is subdominant at low energies compared to the derivative-free measure contribution. At leading order, one can then focus on the functional-measure correction, which is our main interest in this paper.

Note that each side of Equation (1) has its own functional measure. When using the AdS/CFT correspondence to perform calculations in the gauge theory, one has to take them both into account. However, it was shown in Ref. [56] that the functional measure from the gravity side does not lead to additional corrections for a diagonal spacetime metric. In this paper, we are only interested in the AdS ${\mathrm{AdS}}_{5}$–Schwarzschild background:

(12) $$ds^{2}=-g_{tt}\left(u\right)dt^{2}+g_{uu}\left(u\right)du^{2}+g_{xx}\left(u\right)\left(dx^{2}+dy^{2}+dz^{2}\right),$$

with

(13) $$\begin{array}{c} g_{tt}\left(u\right)=\frac{L^{2}h\left(u\right)}{u^{2}},\;g_{uu}\left(u\right)=\frac{L^{2}}{u^{2}h\left(u\right)},\;\;g_{xx}\left(u\right)=\frac{L^{2}}{u^{2}}, \end{array}$$

where $h\left(u\right)=1-u^{4}/u_{0}^{4}$ and $u_{0}$ is the event horizon. We have conveniently set all dimensionful parameters to unity. Because Equation (12) is diagonal, only the functional measure on the gauge side will yield non-trivial contributions, which we calculate in the next section. We emphasize that the measures from each side of the correspondence are not dual to each other. In general (for non-diagonal metrics), calculations in the gauge theory would be affected by both measures: the one defined on the gauge theory itself and the one mapped from the gravity side.

## 3. Response and Transport Coefficients and Quantum Corrections Due to the Functional Measure

In quantum mechanics, perturbation theory sets in by considering a perturbation of a free Hamiltonian $H=H_{\mathrm{FREE}}+\delta H\left(t\right)$, with

(14) $$\begin{array}{cc} \delta H(t) & =-\int d^{3}x\;O\left(\vec{x}\right)\varphi^{\left(0\right)}\left(t,\vec{x}\right). \end{array}$$

External sources $\varphi^{\left(0\right)}$ encode the way the operator O responds to perturbations. The operator’s expected value can be expressed as $\langle O\left(t,\vec{x}\right)\rangle =\mathrm{tr}\;\rho \left(t\right)O\left(\vec{x}\right)$, where ρ(t) stands for the density matrix associated with a canonical ensemble. When the source $\varphi^{\left(0\right)}$ is turned on, the density matrix evolves, and the perturbation of the operator can be written as [57]

(15) $$\begin{array}{c} \delta \langle O(t,\vec{x})\rangle =-i\int d^{4}r^{\prime }G_{R}^{OO}\left(t-t,\vec{x}-{\vec{x}}^{\;\prime }\right)\varphi^{\left(0\right)}\left(t,{\vec{x}}^{\;\prime }\right), \end{array}$$

where the retarded Green’s response function reads

(16) $$\begin{array}{c} G_{R}^{OO}\left(t-t,\vec{x}-{\vec{x}}^{\;\prime }\right)=-i\theta \left(t-t\right)\langle [O\left(t,\vec{x}\right),O\left(t,{\vec{x}}^{\;\prime }\right)]\rangle , \end{array}$$

whose Fourier transformation can be written as $\delta \langle O\left(q\right)\rangle =-\;G_{R}^{OO}\left(q\right)\varphi^{\left(0\right)}\left(q\right)$, where $q_{\mu }=\left(\omega ,\vec{q}\right)$. The response function can therefore be written as

(17) $$\begin{array}{c} G_{R}^{OO}\left(q\right)=-i\int d^{4}r\;e^{i\omega t-i\vec{q}\cdot \vec{x}}\theta \left(t\right)\langle \left[O\left(t,\vec{x}\right),O\left(0,\vec{0}\right)\right]\rangle . \end{array}$$

Equation (17) can be computed forthwith using AdS/CFT tools. In the context of fluid/gravity correspondence, any given Lagrangian can be perturbed as

(18) $$\begin{array}{c} \delta L=h_{\mu \nu }\left(t\right)T^{\mu \nu }\left(\vec{r}\right), \end{array}$$

where the perturbation of the energy-momentum tensor is read off the 2-point retarded Green function acting on the metric perturbation as

(19) $$\begin{array}{ccc} \delta \langle T^{\mu \nu }\rangle & = & -G_{R}^{\mu \nu ,\rho \sigma }h_{\rho \sigma }\left(t\right), \end{array}$$

where

(20) $$\begin{array}{ccc} G_{R}^{\mu \nu ,\rho \sigma } & = & -i\int d^{4}r\;e^{i\omega t-i\vec{q}\cdot \vec{x}}\theta \left(t\right)\langle [T^{\mu \nu }\left(t,\vec{x}\right),T^{\rho \sigma }\left(0,\vec{0}\right)]\rangle \;. \end{array}$$

The energy-momentum tensor couples to metric perturbations in the boundary gauge theory. For the action (9), the effective energy-momentum tensor can be expressed as

(21) $$\begin{array}{cc} T_{\mu \nu }^{\mathrm{EFF}}\; & =\frac{2}{\sqrt{-g^{\left(0\right)}}}\left[\frac{\partial \left(\sqrt{-g^{\left(0\right)}}L_{\mathrm{EFF}}\right)}{\partial {g^{\left(0\right)}}^{\mu \nu }}-\partial_{\rho }\frac{\partial \left(\sqrt{-g^{\left(0\right)}}L_{\mathrm{EFF}}\right)}{\partial \left(\partial_{\rho }{g^{\left(0\right)}}^{\mu \nu }\right)}\right]\\ \; & =T_{\mu \nu }+i\gamma \left(2+\mathrm{tr}\ln\left|g_{\rho \sigma }^{\left(0\right)}\right|\right)g_{\mu \nu }^{\left(0\right)}\;, \end{array}$$

carrying an extra term that encodes quantum corrections due to the functional measure. The arbitrary metric $g_{\mu \nu }^{\left(0\right)}$ has been considered arbitrary thus far. From now on, off-diagonal perturbations of a flat-space approach will be taken into account.

### 3.1. Perfect Fluid Flows in Hydrodynamics

Hydrodynamic fluid flows can be described by the energy-momentum tensor containing quantum corrections, with the conservation law

(22) $$\nabla^{\mu }T_{\mu \nu }^{\mathrm{EFF}}=0$$

compatible with the invariance of the path integral under diffeomorphisms. In this scenario, perfect fluids are governed by the following constitutive relation:

(23) $$\begin{array}{c} {\left(T^{\mathrm{EFF}}\right)}^{\mu \nu }=\left(\epsilon^{\mathrm{EFF}}+P^{\mathrm{EFF}}\right)u^{\mu }u^{\nu }+P^{\mathrm{EFF}}{g^{\left(0\right)}}^{\mu \nu }, \end{array}$$

where $u^{\mu }\left(x\right)$ denotes the 4-velocity and $P^{\mathrm{EFF}}$ stands for the effective pressure, whereas $\epsilon^{\mathrm{EFF}}$ is the fluid energy density. The quantum corrections carried by the effective energy-momentum tensor in Equation (21) induce (23) to yield [42]

(24) $$\begin{array}{cc} \epsilon^{\mathrm{EFF}}\; & =\epsilon -2i\gamma \;, \end{array}$$

(25) $$\begin{array}{cc} P^{\mathrm{EFF}}\; & =P+2i\gamma \;, \end{array}$$

where ϵ and *P* denote, respectively, the energy density and the pressure in relativistic hydrodynamics without quantum corrections due to the functional measure. The imaginary part of Equation (24) points to the instability of degrees of freedom in the fluid and measures its lifetime. The magnitude of this instability is driven by the running parameter γ, whose dependence on the temperature will be analyzed for a non-conformal strongly coupled plasma, in the probe limit and $g_{YM}\gg 1$, with $g_{YM}^{2}N_{c}\gg 1$.

### 3.2. Viscous Fluid Flows

When viscous fluids are taken into account, the constitutive Equation (23) must be replaced by a generalization that includes first-order derivatives of the 4-velocity:

(26) $$\begin{array}{cc} {\left(T^{\mathrm{EFF}}\right)}^{\mu \nu } & =\left(\epsilon^{\mathrm{EFF}}+P^{\mathrm{EFF}}\right)u^{\mu }u^{\nu }+g^{(0)\mu \nu }P^{\mathrm{EFF}}-\Pi^{\mu \rho }\Pi^{\nu \sigma }\left[\eta^{\mathrm{EFF}}\left(\nabla_{(\rho }u_{\sigma )}-\frac{2}{3}g_{\rho \sigma }^{\left(0\right)}\nabla \cdot \vec{u}\right)+\zeta^{\mathrm{EFF}}g_{\rho \sigma }^{\left(0\right)}\nabla \cdot \vec{u}\right], \end{array}$$

where $\Pi^{\mu \nu }:=g^{\left(0\right)\mu \nu }+u^{\mu }u^{\nu }$ denotes the projection on the spatial directions. We denote by $\zeta^{\mathrm{EFF}}$ and $\eta^{\mathrm{EFF}}$ the effective bulk and shear viscosities, respectively. The bulk viscosity measures the mean free path involving any process in which the particle number is not conserved. The shear viscosity appears in the response of the energy-momentum tensor to a small, slowly varying metric perturbation,

(27) $$\begin{array}{c} g_{\mu \nu }^{\left(0\right)}dx^{\mu }dx^{\nu }={\bar{g}}_{\mu \nu }dx^{\mu }dx^{\nu }+2h_{xy}^{\left(0\right)}\left(t\right)dxdy. \end{array}$$

The perturbation of the dissipative term

(28) $$\begin{array}{c} \tau^{xy}:=-\Pi^{x\rho }\Pi^{y\sigma }\left[\eta^{\mathrm{EFF}}\left(\nabla_{(\rho }u_{\sigma )}-\frac{2}{3}g_{\rho \sigma }^{\left(0\right)}\nabla \cdot \vec{u}\right)+\zeta^{\mathrm{EFF}}g_{\rho \sigma }^{\left(0\right)}\nabla \cdot \vec{u}\right] \end{array}$$

in Equation (26) can be evaluated at linear order. The fact that $\nabla_{x}u_{y}=\frac{1}{2}\partial_{t}h_{xy}^{\left(0\right)}$ and the gradient term $\nabla \cdot \vec{u}$ carries second-order terms in the $h_{\mu \nu }$ perturbation implies that

(29) $$\begin{array}{c} \delta \langle \tau^{xy}\rangle =-\eta^{\mathrm{EFF}}\partial_{t}h_{xy}^{\left(0\right)}, \end{array}$$

whose Fourier transform can be written as

(30) $$\begin{array}{c} \lim_{q\to 0}\delta \langle \tau^{xy}\left(\omega ,\vec{q}\right)\rangle =i\omega \eta^{\mathrm{EFF}}h_{xy}^{\left(0\right)}\;. \end{array}$$

A comparison of Equations (19) and (30) yields Kubo’s formula

(31) ηEFF=−limω→0q→01ωImGRxy,xy(ω,q→).

Analogously, the retarded Green’s response function in Equation (20) gives the bulk viscosity [36,58,59]:

(32) ζEFF=limω→0q→01ωImGRPP(ω,q→),

where

(33) $$\begin{array}{ccc} G_{R}^{PP}\left(\omega ,\vec{q}\right) & = & \frac{k_{i}k_{j}k_{m}k_{n}}{k^{4}}\left[G_{R}^{ij,mn}\left(\omega ,\vec{q}\right)+\frac{1}{3}\delta_{ab}T^{ab}\left(\delta^{im}\delta^{jn}+\delta^{in}\delta^{jm}-\delta^{ij}\delta^{mn}\right)\right]+\frac{1}{3}\delta_{ij}T^{ij}\\ & & -\frac{4}{3}G_{R}^{xy,xy}\left(\omega ,\vec{q}\right) \end{array}$$

is the response function to longitudinal fluctuations. When the perturbation (27) is implemented in Equation (21), it yields corrections to the energy-momentum tensor due to the functional measure, resulting in the effective energy-momentum tensor given by

(34) $$T_{\mu \nu }^{\mathrm{EFF}}=T_{\mu \nu }+2i\gamma \eta_{\mu \nu }\;.$$

One can now read off the effective bulk and shear viscosities from Equations (31), (32), and (34) as

(35) $$\begin{array}{cc} \zeta^{\mathrm{EFF}}\; & =\zeta +4i\gamma , \end{array}$$

(36) $$\begin{array}{cc} \eta^{\mathrm{EFF}}\; & =\eta \;. \end{array}$$

The effective shear viscosity does not carry any quantum gravity corrections in the setup considering the functional measure, at least when flat-space backgrounds are regarded. However, the effective bulk viscosity does carry quantum gravity corrections [42]. Equation (21) evinces no corrections for diagonal metrics, and only curved background metrics with non-diagonal terms induce quantum gravity corrections to the shear viscosity. The entropy density at leading order also carries no quantum corrections, since they cancel out those to the effective pressure,

(37) $$\begin{array}{c} s^{\mathrm{EFF}}=\frac{P^{\mathrm{EFF}}+\epsilon^{\mathrm{EFF}}}{T}=s. \end{array}$$

It yields the Kovtun–Son–Starinets result [60] to be invariant in the functional-measure approach:

(38) $$\begin{array}{c} \frac{\eta^{\mathrm{EFF}}}{s^{\mathrm{EFF}}}=\frac{\eta }{s}. \end{array}$$

## 4. Second-Order Derivative Expansion of Relativistic Hydrodynamics

Transport and response coefficients in the second-order hydrodynamical formulation can carry significant signatures of quantum gravity, arising from the presence of a functional measure. One can calculate these coefficients in the N=4 super-Yang–Mills dual plasma [61,62,63,64]. Second-order hydrodynamics provides relevant tools to probe nuclear collisions in the ultrarelativistic limit, making the effective energy density significant for the deconfinement of hadronic matter into the QGP [65,66,67,68,69,70,71].

In the second-order hydrodynamics setup, the dissipative part of the energy-momentum tensor reads [39]

(39) $$\begin{array}{ccc} \Psi^{\mu \nu } & = & -\eta \sigma^{\mu \nu }-\eta \tau_{\pi }\left[u_{\rho }\nabla^{\rho ⌞}\sigma^{\mu \nu ⌟}+\frac{1}{2}\sigma^{\mu \nu }\left(\nabla \cdot \vec{u}\right)\right]+\kappa \left[R^{⌞\mu \nu ⌟}-2u_{\alpha }R^{\alpha ⌞\mu \nu ⌟\beta }u_{\beta }\right]\\ & & +\frac{\lambda_{1}}{\eta^{2}}{\sigma^{⌞\mu }}_{\lambda }\sigma^{\nu ⌟\lambda }-\frac{\lambda_{2}}{\eta }(\Psi {{)}^{⌞\mu }}_{\lambda }\Omega^{\nu ⌟\lambda }+\lambda_{3}{\Omega^{⌞\mu }}_{\lambda }\Omega^{\nu ⌟\lambda }+2\kappa^{*}\;u_{\rho }u_{\sigma }R^{\rho ⌟\mu \nu ⌞\sigma }\\ & & -3\eta \tau_{\pi }\left(\frac{1}{3}-c_{s}^{2}\right)\;\sigma^{\mu \nu }\;\frac{1}{3}\nabla_{\rho }u^{\rho }+\lambda_{4}\left(\nabla^{⌟\mu }\ln s\right)\left(\nabla^{\nu ⌞}\ln s\right)\;, \end{array}$$

involving the Riemann and Ricci tensors and the traceless transverse tensor $\sigma^{\mu \nu }=2^{⌟}\nabla^{\mu }u^{\nu ⌞}$, where the notation

(40) Aμν⌞⌟=−13ΠμνΠαβAαβ+12ΠμαΠνβA(αβ)

for an arbitrary second-rank tensor $A^{\mu \nu }$ is adopted. The symmetrization symbol $A_{\left(\alpha \beta \right)}=\frac{1}{2!}\left(A_{\alpha \beta }-A_{\beta \alpha }\right)$ is also implicit. The vorticity is given by

(41) $$\Omega^{\mu \nu }=\frac{1}{2}\Pi^{\mu \theta }\Pi^{\nu \tau }\left(\nabla_{\theta }u_{\tau }-\nabla_{\tau }u_{\theta }\right)\;.$$

The $\lambda_{1}$, $\lambda_{2}$, $\lambda_{3}$, and $\lambda_{4}$ parameters in Equation (39) are the transport coefficients in second-order hydrodynamics [71].

The response coefficient

(42) $$\begin{array}{c} \kappa =\lim_{\begin{array}{c} q\to 0\\ \omega \to 0 \end{array}}\frac{\partial^{2}}{\partial q^{2}}G_{R}^{xy,xy}\left(\omega ,\vec{q}\right) \end{array}$$

is the gravitational susceptibility of a QGP and enters the expression of the relaxation time for the shear relaxation of fluids with viscosity, which can be written as [39,63,72,73,74]

(43) $$\begin{array}{c} \tau_{\pi }=\frac{1}{2\eta }\left(\lim_{\begin{array}{c} q\to 0\\ \omega \to 0 \end{array}}\frac{\partial^{2}}{\partial \omega^{2}}G_{R}^{xy,xy}\left(\omega ,\vec{q}\right)-\kappa +T\frac{d\kappa }{dT}\right). \end{array}$$

As a thermodynamic coefficient, κ can be computed from lattice QCD [75].

One can derive Kubo’s formulæ for the second-order hydrodynamic coefficients, considering a uniform system in equilibrium at an initial state in Minkowski flat space, introducing perturbatively weak and slowly varying nonuniformity. Again, writing the metric as the sum of the Minkowski metric and a perturbation, $h_{\mu \nu }\left(x\right)$, the metric perturbation tensor couples to the energy-momentum tensor $T^{\mu \nu }$ as, e.g., in Equation (18), generating the expansion in the correlation functions of multiple energy-momentum tensors. The associated coefficients in this expansion consist of the response of the energy-momentum to fluid nonuniformity. One can regard the expectation value $\langle T^{\mu \nu }\left(0\right)\rangle$ for a system in equilibrium at an initial time $t_{0}$ at temperature *T* and consider the metric perturbation $h_{\mu \nu }\left(x\right)$, with $h_{\mu \nu }\left(t\right)\equiv 0$ for all $tl.e.t_{0}$. The energy-momentum tensor reads

(44) $$\begin{array}{ccc} \langle T^{\mu \nu }\left(0\right)\rangle & = & \mathrm{Tr}\;e^{-\beta H}\bar{T}\;\mathrm{EXP}\left(\int_{t_{0}}^{0}dt^{\prime }iH\left[h\left(t^{\prime }\right)\right]\right)\left(T^{\mu \nu }\right)T\;\mathrm{EXP}\left(\int_{t_{0}}^{0}dt^{\prime \prime }\left(-i\right)H\left[h\left(t^{\prime \prime }\right)\right]\right) \end{array}$$

where $\bar{T}\;\mathrm{EXP}$ [TEXP] represents the anti-time-ordered [time-ordered] exponential operators, H[h(t)] is the metric-dependent Hamiltonian, and β is the inverse of the temperature [40,76,77]. Independent metric perturbations of the T-ordered and $\bar{T}$-ordered evolution operators can be introduced to construct the generating functional

(45) $$\begin{array}{ccc} W[h_{1},h_{2}] & \equiv & \ln\mathrm{Tr}\;e^{-\beta H}\bar{T}\mathrm{EXP}\left(i\int_{t_{0}}^{\infty }dt^{\prime }H\left[h_{2}\left(t^{\prime }\right)\right]\right)T\;\mathrm{EXP}\left(-i\int_{t_{0}}^{\infty }dt^{\prime }H\left[h_{1}\left(t^{\prime }\right)\right]\right). \end{array}$$

One then defines the average metric perturbation,

(46) $$\begin{array}{c} h_{\mathrm{AV}}=\frac{1}{2}\left(h_{1}+h_{2}\right) \end{array}$$

and the average energy-momentum tensor $T_{\mathrm{AV}}=\frac{1}{2}\left(T_{1}+T_{2}\right)$, as well as the variables $h_{a}=h_{1}-h_{2}$ and $T_{a}=T_{1}-T_{2}$. Variation with respect to $h_{a}$ yields

(47) $$\langle T_{\mathrm{AV}}^{\mu \nu }\left(x\right)\rangle =\frac{-2i}{\sqrt{-g}}\frac{\partial W}{\partial {\left(h_{a}\right)}_{\mu \nu }\left(x\right)}.$$

After taking the derivative with respect to $h_{a}$, one can set it to zero, implying that $h_{\mathrm{AV}}=h$, as the background $h_{1}=h_{2}=h_{\mathrm{AV}}=h$ remains the case of interest, although considering the term $h_{a}\neq 0$ encodes quantum fluctuations in the metric. One therefore obtains

(48) $$\begin{array}{ccc} {\langle T_{\mathrm{AV}}^{\mu \nu }\rangle }_{h} & = & {\langle T^{\mu \nu }\rangle }_{h=0}-\frac{1}{2}\int d^{4}xG_{ra}^{\mu \nu ,\sigma \rho }\left(0,x\right)h_{\sigma \rho }\left(x\right)\\ & & +\frac{1}{8}\int d^{4}xd^{4}y\;G_{raa}^{\mu \nu ,\sigma \rho ,\tau \alpha }\left(0,x,y\right)h_{\sigma \rho }\left(x\right)h_{\tau \alpha }\left(y\right), \end{array}$$

up to terms of order $O(h_{\mu \nu }^{3})$, for the retarded correlation function, reading [40,71]

(49) $$\begin{array}{c} \;G_{sa\ldots }^{\mu \nu ,\alpha \beta ,\ldots }\left(0,x,\ldots \right)=\lim_{g_{\mu \nu }\to \eta_{\mu \nu }}\frac{{\left(-1\right)}^{n-1}2^{n}i\;\partial^{n}W}{\partial {\left(g_{a}\right)}_{\mu \nu }\left(0\right)\partial {\left(g_{r}\right)}_{\alpha \beta }\left(x\right)\ldots }={\left(-i\right)}^{n-1}\langle T_{\mathrm{AV}}^{\mu \nu }\left(0\right)T_{a}^{\alpha \beta }\left(x\right)\ldots \rangle . \end{array}$$

Equations (42) and (43) hold for both conformal and non-conformal fluids. The energy-momentum tensor in momentum space reads [61]

(50) $$\begin{array}{ccc} {\langle T^{\mu \nu }\left(q\right)\rangle }_{h} & = & {\langle T^{\mu \nu }\rangle }_{h=0}-\frac{1}{2}\int d^{4}q_{1}\delta^{4}\left(q-q_{1}\right)G^{\mu \nu ,\sigma \rho }\left(q;-q_{1}\right)h_{\sigma \rho }\left(q_{1}\right)\\ & & +\frac{1}{8}\int d^{4}q_{1}\int d^{4}q_{2}\delta^{4}\left(q-q_{1}-q_{2}\right)G^{\mu \nu ,\sigma \rho ,\tau \alpha }\left(q;-q_{1},-q_{2}\right)h_{\sigma \rho }\left(q_{1}\right)h_{\tau \alpha }\left(q_{2}\right)+\ldots \end{array}$$

Assuming that $q_{1}^{\mu }=\left(\omega_{1},0,0,q_{1}\right)$ and $q_{2}^{\mu }=\left(\omega_{2},0,0,q_{2}\right)$, the transport coefficients $\lambda_{1},\lambda_{2}$, and $\lambda_{3}$ can be read off from 3-point correlators in the strong-coupling regime when evaluating the cubic Witten diagrams as [7,61]

(51) $$\begin{array}{ccc} & & \lim_{\begin{array}{c} \omega_{1}\to 0\\ \omega_{2}\to 0 \end{array}}\frac{\partial }{\partial \omega_{2}}\frac{\partial }{\partial \omega_{1}}\lim_{\begin{array}{c} q_{2}\to 0\\ q_{1}\to 0 \end{array}}G^{xy,zx,yz}=\eta \tau_{\pi }-\lambda_{1},\\ & & \lim_{\begin{array}{c} \omega_{1}\to 0\\ q_{2}\to 0 \end{array}}\frac{\partial }{\partial \omega_{1}}\frac{\partial }{\partial q_{2}}\lim_{\begin{array}{c} q_{2}\to 0\\ \omega_{1}\to 0 \end{array}}G^{xy,yz,x0}=\frac{1}{2}\eta \tau_{\Pi }-\frac{1}{4}\lambda_{2},\\ & & \lim_{\begin{array}{c} q_{1}\to 0\\ q_{2}\to 0 \end{array}}\frac{\partial }{\partial q_{2}}\frac{\partial }{\partial q_{1}}\lim_{\begin{array}{c} \omega_{1}\to 0\\ \omega_{2}\to 0 \end{array}}G^{xy,x0,y0}=-\frac{1}{4}\lambda_{3}, \end{array}$$

where $\tau_{\Pi }$ is the bulk relaxation time. Throughout this work, after Equation (13), we put all dimensionful parameters to unity. For completeness, we restore the event horizon $u_{0}$ from here on. Let us first discuss the coefficient κ in Equation (42) by defining the regularized quantity

(52) $$\begin{array}{c} \kappa_{\varepsilon }:=-\lim_{\begin{array}{c} q\to 0\\ \omega \to 0 \end{array}}\frac{\partial^{2}}{\partial q^{2}}F\left(\omega ,q;\varepsilon \right)=\frac{1}{2\varepsilon^{2}}+\int_{u_{0}}^{\varepsilon }du\sqrt{-g^{\left(0\right)}}g^{xx}\left(u\right). \end{array}$$

Also, the expression that follows lacks any ultraviolet divergence [63]:

(53) $$\begin{array}{ccc} \kappa (T)= & \lim_{\varepsilon \to 0}\left(\kappa_{\varepsilon }\left(T\right)-\kappa_{\varepsilon }\left(T_{\mathrm{HIGH}}\right)\right)+\frac{T_{\mathrm{HIGH}}^{2}N_{c}^{2}}{8}-\kappa_{0} & \\ \approx & \lim_{\varepsilon \to 0}\left[\int_{u_{0}\left(T\right)}^{\varepsilon }du\;\sqrt{-g^{\left(0\right)}}g^{xx}\left(u\right){|}_{u_{0}\left(T\right)}-\int_{u_{\mathrm{HIGH}}}^{\varepsilon }du\;\sqrt{-g^{\left(0\right)}}g^{xx}\left(u\right){|}_{u_{\mathrm{HIGH}}}\right]+\frac{T_{high}^{2}N_{c}^{2}}{8}-\kappa_{0}\;, & \end{array}$$

where $T_{\mathrm{HIGH}}$ is a sufficiently large temperature, which implies proximity to the ultraviolet fixed point. The temperature-dependent part, which is independent of the ultraviolet cutoff ε of $\kappa_{\varepsilon }\left(T_{\mathrm{HIGH}}\right)$, approaches $\kappa_{\mathrm{SYM}}\left(T_{\mathrm{HIGH}}\right)$, $u_{\mathrm{HIGH}}=u_{0}\left(T_{\mathrm{HIGH}}\right)$, and $\kappa_{0}$ is a constant to be subtracted to ensure that $\kappa (T_{\mathrm{MIN}})=0$, where $T_{\mathrm{MIN}}$ is the lowest temperature considered in the numerical calculations ($T_{\mathrm{MIN}}∼10$ MeV) [63]. Analogously, to evaluate the relaxation time for the shear relaxation $\tau_{\pi }$ in Equation (43), one can define

(54) $$\begin{array}{cc} \tau_{\pi } & =\frac{1}{2\eta }\left(\Omega -\kappa +T\frac{d\kappa }{dT}\right), \end{array}$$

and

(55) $$\begin{array}{cc} \Omega_{\varepsilon } & :=\lim_{\begin{array}{c} q\to 0\\ \omega \to 0 \end{array}}\frac{\partial^{2}}{\partial \omega^{2}}F\left(\omega ,q;\varepsilon \right)=\frac{L^{3}}{2\varepsilon^{2}}+g_{xx}^{3/2}\left(u_{0}\right)\int_{\varepsilon }^{u_{0}}du\left(\frac{g_{xx}^{3/2}\left(u_{0}\right)}{\sqrt{-g^{\left(0\right)}}g^{uu}\left(u\right)}-\frac{\sqrt{-g^{\left(0\right)}}g^{tt}\left(u\right)}{g_{xx}^{3/2}\left(u_{0}\right)}\right), \end{array}$$

Now, the UV-finite expression can be evaluated as

(56) $$\begin{array}{cc} \Omega (T) & =\lim_{\varepsilon \to 0}\left(\Omega_{\varepsilon }\left(T\right)-\Omega_{\varepsilon }\left(T_{\mathrm{HIGH}}\right)\right)+\Omega_{\mathrm{SYM}}\left(T_{\mathrm{HIGH}}\right)-\Omega_{0}\\ \; & ≊\lim_{\varepsilon \to 0}\left(g_{xx}^{3/2}\left(u_{0}\right)\int_{\varepsilon }^{u_{0}\left(T\right)}du\left[\frac{g_{xx}^{3/2}\left(u_{0}\right)}{\sqrt{-g^{\left(0\right)}}g^{uu}\left(u\right)}-\frac{\sqrt{-g^{\left(0\right)}}g^{tt}\left(u\right)}{g_{xx}^{3/2}\left(u_{0}\right)}\right]{|}_{u_{0}\left(T\right)}\right.\\ \; & \left.\;-g_{xx}^{3/2}\left(u_{\mathrm{HIGH}}\right)\int_{\varepsilon }^{u_{\mathrm{HIGH}}}du\left[\frac{g_{xx}^{3/2}\left(u_{\mathrm{HIGH}}\right)}{\sqrt{-g^{\left(0\right)}}g^{uu}\left(u\right)}-\frac{\sqrt{-g^{\left(0\right)}}g^{tt}\left(u\right)}{g_{xx}^{3/2}\left(u_{\mathrm{HIGH}}\right)}\right]{|}_{u_{\mathrm{HIGH}}}\right)+\frac{T_{\mathrm{HIGH}}^{2}N_{c}^{2}}{8}\left[1-\ln2\right], \end{array}$$

up to a finite constant that must be subtracted to ensure that $\tau_{\pi }\left(T_{\mathrm{MIN}}\right)\eta \left(T_{\mathrm{MIN}}\right)=0$. These integrals can be evaluated for ε=0.02 [63].

For the conformal super-Yang–Mills plasma, some of the following transport coefficients

(57) $$\begin{array}{ccc} \tau_{\pi } & = & \frac{2-\ln2}{2\pi T}, \end{array}$$

(58) $$\begin{array}{ccc} \lambda_{1} & = & \frac{N_{c}^{2}T^{2}}{16}, \end{array}$$

(59) $$\begin{array}{ccc} \lambda_{2} & = & -\frac{\ln2}{8}N_{c}^{2}T^{2}, \end{array}$$

(60) $$\begin{array}{ccc} \lambda_{3} & = & 2\kappa -T\frac{d\kappa }{dT}, \end{array}$$

(61) $$\begin{array}{ccc} \kappa & = & \frac{T^{2}N_{c}^{2}}{8}, \end{array}$$

receive corrections from quantum gravity, encoded into the running parameter γ, where *T* denotes the temperature of the QCD system. Such corrections, introduced by the functional measure, can be taken into account when Equation (39) is promoted to

(62) $$\begin{array}{ccc} {\left(\Psi^{\mathrm{EFF}}\right)}^{\mu \nu } & = & -\eta^{\mathrm{EFF}}\sigma^{\mu \nu }-\eta^{\mathrm{EFF}}\tau_{\pi }^{\mathrm{EFF}}\left[u_{\rho }\nabla^{\rho ⌞}\sigma^{\mu \nu ⌟}+\frac{1}{2}\sigma^{\mu \nu }\left(\nabla \cdot \vec{u}\right)\right]+\kappa^{\mathrm{EFF}}\left[R^{⌞\mu \nu ⌟}-2u_{\alpha }R^{\alpha ⌞\mu \nu ⌟\beta }u_{\beta }\right]\\ & & +\frac{\lambda_{1}^{\mathrm{EFF}}}{{\left(\eta^{\mathrm{EFF}}\right)}^{2}}{\sigma^{⌞\mu }}_{\rho }\sigma^{\nu ⌟\rho }-\frac{\lambda_{2}^{\mathrm{EFF}}}{\eta^{\mathrm{EFF}}}{\sigma^{⌞\mu }}_{\rho }\Omega^{\nu ⌟\rho }+\lambda_{3}^{\mathrm{EFF}}{\Omega^{⌞\mu }}_{\rho }\Omega^{\nu ⌟\rho }+2{\left(\kappa^{*}\right)}^{\mathrm{EFF}}\;u_{\rho }u_{\sigma }R^{\rho ⌟\mu \nu ⌞\sigma }\\ & & -\eta^{\mathrm{EFF}}\tau_{\pi }^{\mathrm{EFF}}\left(\frac{1}{3}-c_{s}^{2}\right)\;\sigma^{\mu \nu }\nabla_{\rho }u^{\rho }+\lambda_{4}\left(\nabla^{⌟\mu }\ln s\right)\left(\nabla^{\nu ⌞}\ln s\right). \end{array}$$

Ref. [42] showed that the speed of sound, appearing in the penultimate term in Equation (62), remains invariant under quantum gravity corrections. The coefficients carrying quantum corrections in the dissipative part of the energy-momentum tensor in Equation (62) can be related to those in Equation (39), which do not have quantum corrections, as

(63) $$\begin{array}{ccc} \tau_{\pi }^{\mathrm{EFF}} & = & \tau_{\pi }+12\frac{\left(2-\ln2\right)}{\pi T}\gamma^{2}, \end{array}$$

(64) $$\begin{array}{ccc} \lambda_{1}^{\mathrm{EFF}} & = & \lambda_{1}+\frac{13N_{c}^{2}T^{2}}{4}\left(13+\gamma^{2}\right)\gamma^{2}, \end{array}$$

(65) $$\begin{array}{ccc} \lambda_{2}^{\mathrm{EFF}} & = & \lambda_{2}-\gamma^{2}\left(6+\gamma^{2}\right)\frac{\ln2}{4}N_{c}^{2}T^{2}, \end{array}$$

(66) $$\begin{array}{ccc} \lambda_{3}^{\mathrm{EFF}} & = & 2\kappa^{\mathrm{EFF}}-T\frac{d\kappa^{\mathrm{EFF}}}{dT}=\lambda_{3}, \end{array}$$

(67) $$\begin{array}{ccc} \kappa^{\mathrm{EFF}} & = & \kappa +\frac{3T^{2}N_{c}^{2}}{4}\gamma^{2}. \end{array}$$

Among the effective response and transport coefficients (63)–(67), only $\lambda_{3}$ does not receive any correction. The temperature scale is chosen to match the minimum speed of sound computed holographically with that found on the lattice results for (2 + 1)-flavor QCD [78]. Ref. [63] obtained the pseudo-critical temperature for the chiral crossover transition $T_{c}=143.8$ MeV, indicating a crossover phase transition from the QGP to hadronic matter. The authors proposed fitting the quantity $\frac{\tau_{\pi }\eta }{T^{2}}$ as a function of $\frac{T}{T_{c}}$, given by

(68) $$\begin{array}{c} \frac{\tau_{\pi }\eta }{T^{2}}=\frac{0.2664}{1+\exp\left[2.029\left(0.7413-\frac{T}{T_{c}}\right)\right]+\exp\left[-0.1717\left(10.76+\frac{T}{T_{c}}\right)\right]+\exp\left[9.763\left(1.074-\frac{T}{T_{c}}\right)\right]}. \end{array}$$

Therefore, the running parameter γ, driving corrections due to a functional measure encoding quantum gravity effects, can be realized as a temperature-dependent parameter. Data obtained from (2+1)-flavor lattice QCD for the pressure and relaxation time can be compared to the results whose quantum gravity corrections were estimated in Section 3 and Section 4 for temperatures in the range 130 MeV ≲T≲450 MeV, where an entirely hadronic description is not appropriate. Additionally, this temperature range is not sufficiently high to ensure a straightforward formulation through perturbative aspects of QCD.

**Figure 1:**
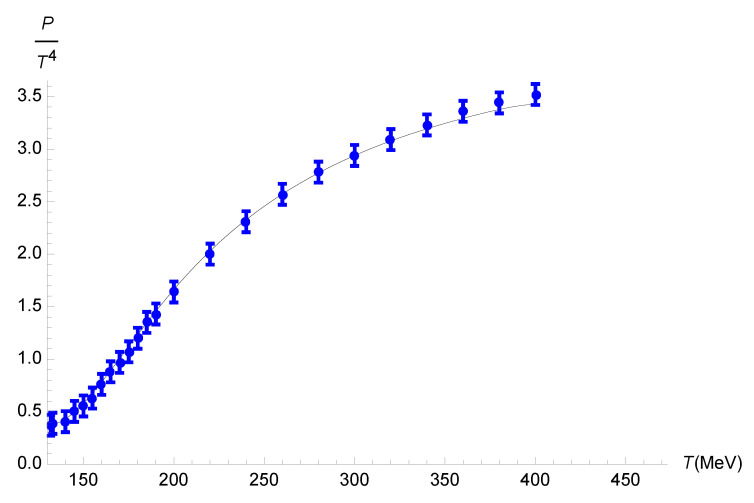
Plot of $P/T^{4}$ as a function of *T* for the bottom-up holographic model, indicated by the black curve. The results for the lattice (2+1)-flavor QCD are plotted as blue points [78].

We first analyze the behavior of the pressure as a function of the temperature, as depicted in Figure 1.

The function $P/T^{4}$ can be interpolated as a polynomial function of the temperature for the bottom-up holographic model as

(69) $$\begin{array}{ccc} \frac{P}{T^{4}} & = & -5.8077\times 10^{-16}\;T^{7}+1.1725\times 10^{-12}\;T^{6}-1.0057\times 10^{-9}\;T^{5}+4.7444\times 10^{-7}\;T^{4}\\ & & -1.3266\times 10^{-4}\;T^{3}+2.1860\times 10^{-2}\;T^{2}-1.9328\;T+70.185, \end{array}$$

within a 0.01% root-mean-square deviation.

Using Equation (25) yields

(70) $$\begin{array}{c} \left|P^{\mathrm{EFF}}\right|=P\sqrt{1+\frac{4\gamma^{2}}{P^{2}}}. \end{array}$$

Using Equation (63) yields an effective relaxation time $\tau_{\pi }^{\mathrm{EFF}}$ encoding quantum gravity effects, which can be related to the standard relaxation time $\tau_{\pi }$ in (57) without quantum corrections. To compute the shear relaxation coefficient, $\eta \tau_{\pi }/T^{2}$, one can alternatively use Equation (15) from Ref. [40] and Equation (48) from Ref. [36], namely

(71) $$\begin{array}{c} \eta \tau_{\pi }=-\frac{1}{2}\lim_{\begin{array}{c} q\to 0\\ \omega \to 0 \end{array}}\frac{\partial^{2}}{\partial \omega^{2}}G_{R}^{xy,xy}\left(\omega ,\vec{q}\right)+\frac{1}{2}\lim_{\begin{array}{c} q\to 0\\ \omega \to 0 \end{array}}\frac{\partial^{2}}{\partial q^{2}}G_{R}^{xy,xy}\left(\omega ,\vec{q}\right). \end{array}$$

**Figure 2:**
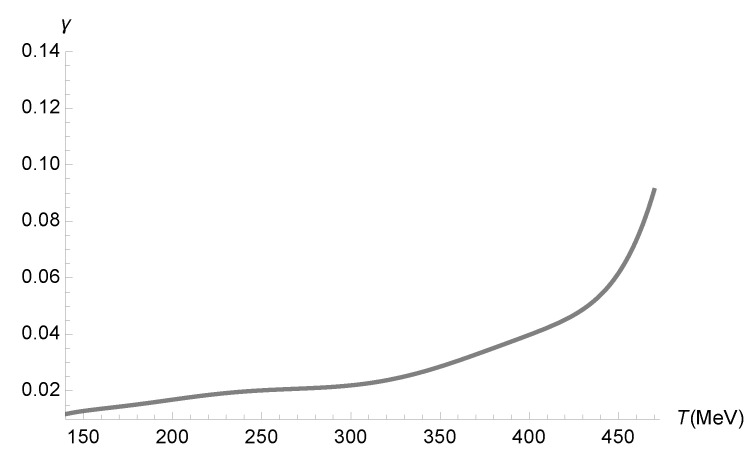
Range of the running parameter γ (${\mathrm{GeV}}^{4}$) as a function of the temperature (MeV), using the numerical data corresponding to Figure 1.

One can therefore employ the data in Figure 1 to obtain the running parameter γ as a function of the temperature in Figure 2. In fact, the QGP carries quantum corrections due to the functional measure. Hence, the left-hand side of Equation (68), which reads off the relationship between the relaxation time and the temperature, regards the quantity $\frac{\tau_{\pi }^{\mathrm{EFF}}\eta }{T^{2}}$. In turn, Equation (63) relates the effective relaxation time, containing quantum corrections, to the relaxation time without quantum corrections. The left-hand side of Equation (68) then yields

(72) $$\begin{array}{c} \frac{\tau_{\pi }\eta }{T^{2}}+12\frac{(2-\ln2)\eta }{\pi T^{3}}\gamma^{2}. \end{array}$$

From it, we can use Equation (57) and solve Equation (68) for the running parameter γ as a function of the temperature.

**Figure 3:**
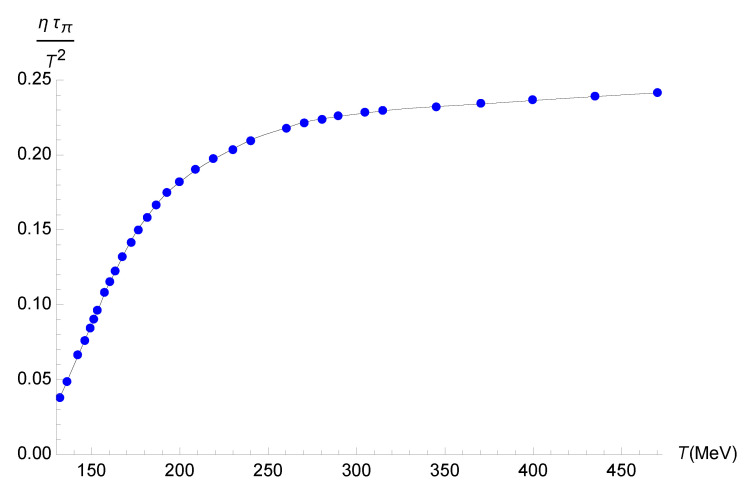
Plot of $\tau_{\pi }\eta /T^{2}$ as a function of *T* for the bottom-up holographic model. The blue points correspond to the numerical data, whereas the black line corresponds to the fit in Equation (68).

Figure 3 can be interpolated as a polynomial function of the temperature for the bottom-up holographic model as

(73) $$\begin{array}{ccc} \frac{\tau_{\pi }\eta }{T^{2}} & = & -3.3890\times 10^{-17}\;T^{7}+7.1330\times 10^{-14}\;T^{6}-6.2508\times 10^{-11}\;T^{5}+2.9417\times 10^{-8}\;T^{4}\\ & & -7.9647\times 10^{-6}\;T^{3}+1.2219\times 10^{-3}\;T^{2}-9.4946\times 10^{-2}T+2.8185, \end{array}$$

within a 0.01% root-mean-square deviation.

**Figure 4:**
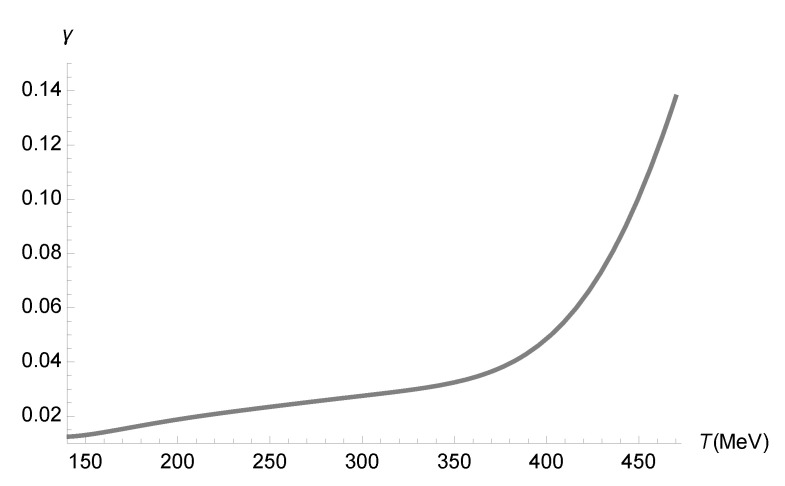
Range of the running parameter γ (${\mathrm{GeV}}^{4}$) as a function of the temperature *T* (MeV), using the numerical data corresponding to Figure 1 and Equation (70).

Now, Equation (70) can be employed, together with Figure 1 to obtain the range of the running parameter γ as a function of the temperature *T*. The results are displayed in Figure 4.

For the running parameter γ=γ(Λ), an energy scale Λ∼ 3.0 TeV can be adopted in Equation (10), as experimental results involving the QGP have been observed in Pb+Pb and p-Pb collisions at $\sqrt{s_{NN}}$ = 2.76 and 5.02 TeV by the ALICE experiment at the LHC.

## 5. Bounding the Parameter γ from Experimental Data from LHC and RHIC

A reliable bound on the parameter γ, which drives the quantum gravity corrections due to the functional measure, can be inferred from experimental data at the LHC and RHIC regarding the ζ/s ratio of the QGP. It can be compared with those whose quantum corrections were predicted in Section 3 and Section 4. The values of the transport coefficients of the QGP have been precisely determined in heavy-ion collision experiments for temperatures in the range 150 MeV ≲T≲350 MeV. The lower limit in this range, at least for zero baryon density or baryon chemical potential, approximately corresponds to the pseudo-critical temperature of a smooth crossover between the confined and the deconfined phase [79,80]. To determine a trustworthy bound on γ, Equation (35) can be employed, denoting

(74) $$\begin{array}{c} \left|\zeta^{\mathrm{EFF}}\right|=\zeta {\left(1+\frac{16\gamma^{2}}{\zeta^{2}}\right)}^{1/2}. \end{array}$$

**Figure 5:**
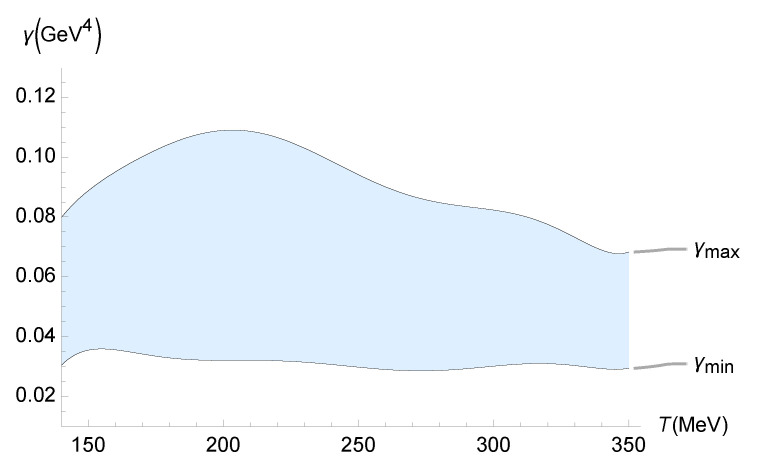
Experimental bound on γ (${\mathrm{GeV}}^{4}$) as a function of the QGP temperature (MeV), using the experimental range of ζ/s determined by the JETSCAPE Bayesian model [49,50].

One can split the analyses of up-to-date experimental estimates for the QGP bulk viscosity into five independent parts. The first one takes into account the JETSCAPE Bayesian model [49,50]. Bayesian inference is employed to obtain probabilistic constraints for ζ/s from experimental and theoretical uncertainties. Bayesian model averaging accounts for the transition from a hydrodynamical fluid describing the QGP to hadronic transport in the final evolution stage, yielding a reliable phenomenological constraint range for ζ/s [81]. An experimental bound $\gamma_{\min}≲\gamma ≲\gamma_{\max}$, for the parameter carrying quantum gravity corrections generated by a functional measure, is depicted in Figure 5 as a function of the QGP temperature.

One can interpolate the QGP temperature-dependent lower and upper bounds on γ in Figure 5, using the polynomials

(75) $$\begin{array}{cc} \gamma_{\mathrm{MIN}}(T)= & 4.47201\times 10^{-16}\;T^{7}-7.82417\times 10^{-13}\;T^{6}+5.78159\times 10^{-10}\;T^{5}-2.33781\times 10^{-7}\;T^{4}\\ & \;\;+5.58432\times 10^{-5}\;T^{3}-7.87737\times 10^{-2}\;T^{2}+0.60739\;T-19.7078, \end{array}$$

(76) $$\begin{array}{cc} \gamma_{\mathrm{MAX}}(T)= & 6.01245\times 10^{-16}\;T^{7}-1.00634\times 10^{-12}\;T^{6}+7.08114\times 10^{-10}\;T^{5}-2.71365\times 10^{-7}\;T^{4}\\ & \;\;+6.11704\times 10^{-5}\;T^{3}-8.12106\times 10^{-3}\;T^{2}+0.58963\;T-18.0404, \end{array}$$

within $10^{-3}\%$ interpolation error.

**Figure 6:**
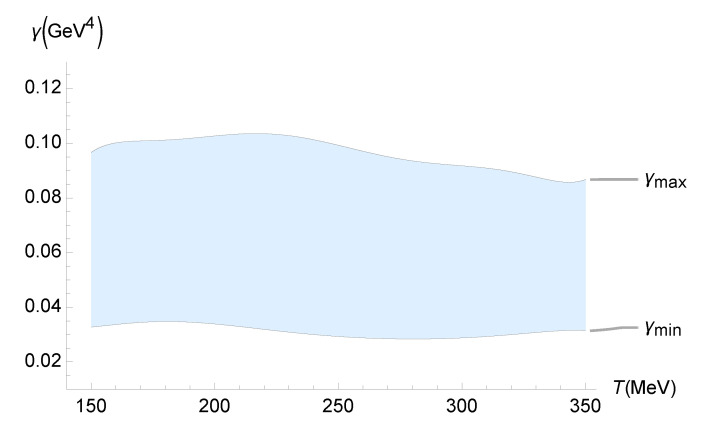
Experimental bounds on γ (${\mathrm{GeV}}^{4}$) as a function of the QGP temperature (MeV), using the experimental range of ζ/s determined by the analysis of the Duke group [51].

The second part of the analysis consists of considering the experimental data on ζ/s of the QGP, as outlined by the Duke group [51]. Their results present very precise estimates for the experimental value of ζ/s for the QGP. They include quantitative uncertainties from Bayesian parameter estimation protocols involving the analysis of a dynamical collision model and experimental data. This time, the QGP temperature-dependent lower and upper bounds on γ are depicted in Figure 6.

These lower and upper bounds on γ in Figure 6 can be, respectively, interpolated by

(77) $$\begin{array}{cc} \gamma_{\mathrm{MIN}}\left(T\right)= & -8.78999\times 10^{-17}\;T^{7}+1.54981\times 10^{-13}\;T^{6}-1.15801\times 10^{-10}\;T^{5}+4.74402\times 10^{-8}\;T^{4}\\ & \;\;-1.14777\times 10^{-5}\;T^{3}+1.63481\times 10^{-2}\;T^{2}-0.126542\;T+4.13012, \end{array}$$

(78) $$\begin{array}{cc} \gamma_{\mathrm{MAX}}(T)= & 6.07662\times 10^{-16}T^{7}-1.06355\times 10^{-12}T^{6}+7.86621\times 10^{-10}T^{5}-3.18494\times 10^{-7}T^{4}\\ & \;\;+7.62074\times 10^{-5}T^{3}-1.07744\times 10^{-2}T^{2}+0.83363\;T-27.1427, \end{array}$$

within $10^{-3}\%$ interpolation error.

**Figure 7:**
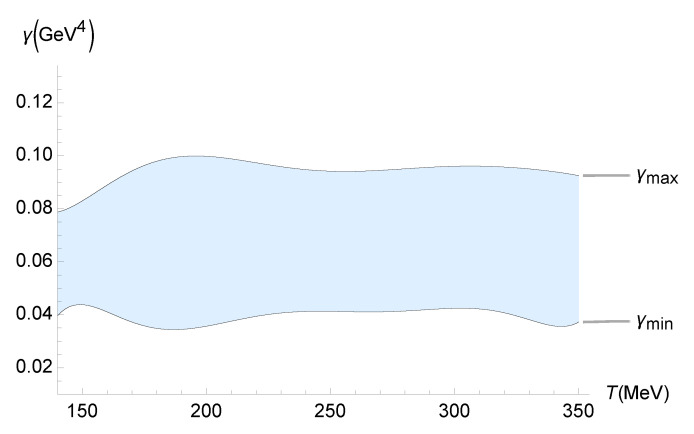
Experimental bounds on γ (${\mathrm{GeV}}^{4}$) as a function of the QGP temperature (MeV), using the experimental range of ζ/s determined by the Jyväskylä–Helsinki–Munich group [52].

The up-to-date experimental results of ζ/s, implemented by the Jyväskylä–Helsinki–Munich group [52], can now be used. They refer, in particular, to ζ/s of the QGP in relativistic heavy-ion collisions, which are quantified through an improved global Bayesian analysis using the Pb-Pb LHC experimental data. The lower and upper bounds on γ, as a function of the QGP temperature, are illustrated in Figure 7.

The upper and lower bounds on γ in Figure 7 can be, respectively, interpolated by the polynomials

(79) $$\begin{array}{cc} \gamma_{\mathrm{MIN}}\left(T\right)= & 8.15869\times 10^{-16}\;T^{7}-1.42094\times 10^{-12}\;T^{6}+1.04585\times 10^{-9}\;T^{5}-4.21334\times 10^{-7}\;T^{4}\\ & \;\;+1.00242\times 10^{-4}T^{3}-0.014069\;T^{2}+1.07749\;T-34.6634, \end{array}$$

(80) $$\begin{array}{cc} \gamma_{\mathrm{MAX}}\left(T\right)= & -1.96075\times 10^{-16}\;T^{7}+3.64874\times 10^{-13}\;T^{6}-2.86110\times 10^{-10}\;T^{5}+1.22239\times 10^{-7}\;T^{4}\\ & \;\;-3.06481\times 10^{-5}\;T^{3}+4.49533\times 10^{-3}\;T^{2}-0.35592\;T+11.7849, \end{array}$$

within $10^{-3}\%$ interpolation error. These results update the ones in Ref. [42].

**Figure 8:**
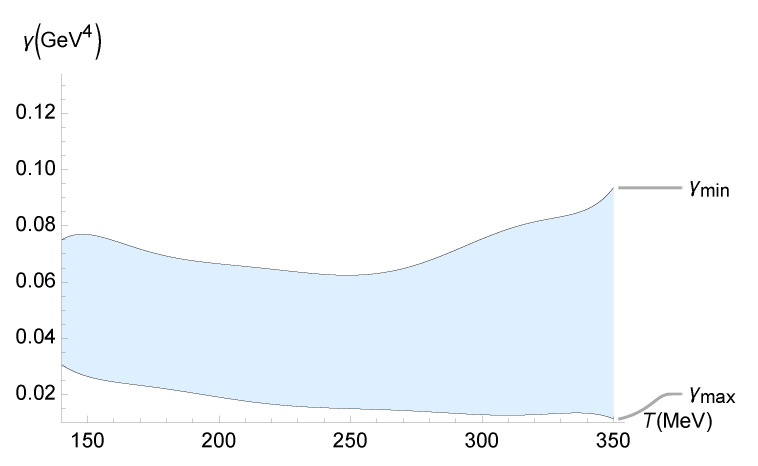
Experimental bound on γ (${\mathrm{GeV}}^{4}$) as a function of the QGP temperature (MeV), using the experimental range of ζ/s determined from the analysis of experimental data of the QGP at LHC by the MIT–Utrecht–Genève group using the Trajectum framework [54].

The experimental data on the ζ/s ratio for the QGP at LHC were analyzed by the MIT–Utrecht–Genève group using the Trajectum setup in an improved global Bayesian survey of the Pb-Pb LHC data at $\sqrt{s_{NN}}=2.76$ and 5.02 TeV. Ref. [54] demonstrated a non-negligible effect of the QGP ζ/s in heavy-ion collision observables. This analysis takes into account the measurements of higher-order harmonics in the hydrodynamical fluid flow, as well as those in the flow fluctuation observables, as inputs in the Bayesian analysis. The QGP temperature-dependent bound on γ is represented in Figure 8.

The lower and upper values of the bound on γ in Figure 8 are, respectively, represented by the following polynomials:

(81) $$\begin{array}{cc} \gamma_{\mathrm{MIN}}\left(T\right)= & 6.57587\times 10^{-16}\;T^{7}-1.11752\times 10^{-12}\;T^{6}+8.01756\times 10^{-10}\;T^{5}-3.14696\times 10^{-7}\;T^{4}\\ & \;\;+7.29776\times 10^{-5}\;T^{3}-9.99738\times 10^{-3}\;T^{2}+0.748838\;T-23.5695, \end{array}$$

(82) $$\begin{array}{cc} \gamma_{\mathrm{MAX}}\left(T\right)= & -2.67183\times 10^{-16}\;T^{7}+4.58546\times 10^{-13}\;T^{6}-3.32588\times 10^{-10}\;T^{5}+1.32076\times 10^{-7}\;T^{4}\\ & \;\;-3.09994\times 10^{-5}\;T^{3}+4.299319\times 10^{-3}\;T^{2}-0.32639\;T+10.4997, \end{array}$$

within $10^{-3}\%$ interpolation error.

**Figure 9:**
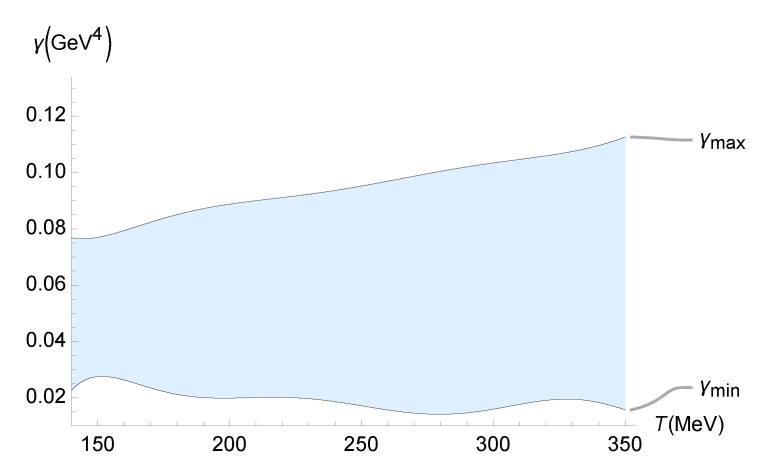
Experimental bounds on γ (${\mathrm{GeV}}^{4}$) as a function of the QGP temperature (MeV), using the experimental range of ζ/s determined by the Shanghai group [55].

Finally, the fifth independent part of this study uses the quark recombination model. The elliptic flows of ϕ and ω mesons, produced in Au+Au collisions at $\sqrt{s_{NN}}=200$ GeV, and in Pb+Pb collisions at $\sqrt{s_{NN}}=2.76$ TeV, are employed [55]. Therefore, the temperature-dependent lower and upper bounds on the γ parameter are plotted in Figure 9.

The QGP temperature-dependent lower and upper bounds on the running parameter γ, depicted in Figure 9, are interpolated by the polynomials

(83) $$\begin{array}{cc} \gamma_{\mathrm{MIN}}\left(T\right)= & 4.94397\times 10^{-16}T^{7}-8.86412\times 10^{-13}T^{6}+6.69641\times 10^{-10}T^{5}-2.76128\times 10^{-7}T^{4}\\ & \;\;+6.70844\times 10^{-5}T^{3}-9.59746\times 10^{-3}\;T^{2}+0.748303\;T-24.4925, \end{array}$$

(84) $$\begin{array}{cc} \gamma_{\mathrm{MAX}}\left(T\right)= & -1.23113\times 10^{-16}T^{7}+2.29331\times 10^{-13}T^{6}-1.79668\times 10^{-10}T^{5}+7.66885\times 10^{-8}T^{4}\\ & \;\;-1.92437\times 10^{-5}T^{3}+2.83603\times 10^{-3}T^{2}-0.226875\;T+7.66334, \end{array}$$

within $10^{-3}\%$ interpolation error.

A final remark is worth discussing, which involves the possibility of expressing the quantum-corrected bulk-viscosity-to-entropy-density ratio as a deformation of the classical bulk-viscosity-to-entropy-density ratio, as

(85) $$\begin{array}{c} \frac{\left|\zeta^{\mathrm{EFF}}\right|}{s^{\mathrm{EFF}}}=\frac{\zeta }{s}\left(1+f(T)\right), \end{array}$$

where f(T) is some temperature-dependent function that regulates quantum corrections. Let us first remember that Equation (37) states that the entropy density does not carry any quantum corrections, as $s^{\mathrm{EFF}}=s.$ Taking this into account, one can reasonably assume that, as quantum corrections are expected to be tiny, the running parameter γ satisfies |γ|≪|ζ|. Therefore, using Equation (74), the bulk-viscosity-to-entropy-density ratio with quantum gravity corrections can be written in terms of the classical bulk-viscosity-to-entropy-density ratio, up to $O(\gamma^{4}/\zeta^{4})$, as

(86) $$\begin{array}{c} \frac{\left|\zeta^{\mathrm{EFF}}\right|}{s^{\mathrm{EFF}}}=\frac{\left|\zeta^{\mathrm{EFF}}\right|}{s}≊\frac{\zeta }{s}\left(1+\frac{8\gamma^{2}}{\zeta^{2}}\right). \end{array}$$

From Equation (86), one can realize that the required function f(T) is given by

(87) $$\begin{array}{c} f\left(T\right)=\frac{8\gamma^{2}\left(T\right)}{\zeta^{2}\left(T\right)}. \end{array}$$

**Figure 10:**
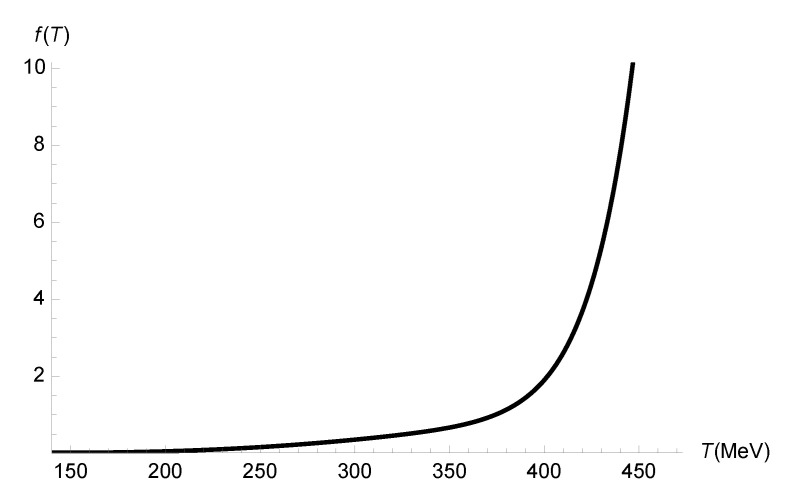
Quantum-correcting function f(T), which drives quantum corrections to the bulk-viscosity-to-entropy-density ratio in Equation (74), as a function of the temperature (MeV).

Ref. [63] showed the bulk-viscosity-to-entropy-density ratio ζ/s as a function of the temperature *T* for the bottom-up holographic model, with a resonance-like fitting function

(88) $$\frac{\zeta }{s}\left(T\right)=\frac{0.01162}{\sqrt{{\left(\frac{T}{T_{c}}-1.104\right)}^{2}+0.05697}}-\frac{0.1081}{\frac{T^{2}}{T_{c}^{2}}+23.716},$$

with $T_{c}=143.8$ MeV. The first term in Equation (88) implements a resonance-like peak, whereas the second term is responsible for a smooth background apart from the peak. We can substitute the temperature-dependent bulk viscosity (88) on the very right-hand side of Equation (86), keeping in mind the expression for the entropy density $s=\pi^{2}N_{c}^{2}T^{3}/2$. The plot of the quantum-correcting function, γ=γ(T), is shown in Figure 10.

Figure 10 indicates that the quantum-correcting function f(T), which drives quantum corrections to the bulk-viscosity-to-entropy-density ratio in Equation (74), increases more sharply for T≳380 MeV. At the pseudo-critical temperature for the chiral crossover transition, $f(T_{c})=0.01578$, representing a 1.57% margin for quantum corrections to the classical ζ/s ratio.

## 6. Discussion

In this paper, we used the gauge/gravity duality to determine the transport coefficients of a non-conformal, strongly interacting non-Abelian plasma. This fluid displays a crossover transition similar to that found in the lattice calculations of the QGP. We computed the contributions to the transport and response coefficients, including those from the second-order expansion, due to the presence of a functional measure in quantum gravity. The comparison of the resulting pressure and relaxation time with existing data from lattice simulations led to the discovery of the temperature dependence of the coupling constant γ, which controls the functional-measure strength. In both cases, we found an upward trend in γ as the temperature increased. Even within the upper-temperature limit of the lattice data used, one finds $\gamma ∼10^{-1}$ GeV^4^. Since large-$N_{c}$ gauge theories can be employed to model QCD, the results obtained in this work can be applied when scrutinizing several other aspects of the QGP. Quantum gravity corrections implemented by a functional measure may be examined, at least in principle, in experiments involving the QGP in the hydrodynamical regime, mainly those regarding the QGP transport coefficients. As shown in Equations (35) and (36), only the ζ/s ratio can detect quantum gravity effects, which are unseen by the η/s ratio. A relevant method was implemented and illustrated in Figure 5, Figure 6, Figure 7, Figure 8 and Figure 9, showing the QGP temperature-dependent lower and upper bounds on the parameter γ, carrying quantum gravity effects on ζ/s. It complements and refines the results already obtained in this work. The analysis relies on the up-to-date experimental results for the ζ/s ratio measured for the QGP [49,50,51,52,53,54,55]. We conclude that the experimental range of the bulk-viscosity-to-entropy density of the QGP, obtained by five different phenomenological analyses (JETSCAPE Bayesian model, Duke, Jyväskylä–Helsinki–Munich, MIT–Utrecht–Genève, and Shanghai), corroborates the existence of a non-vanishing renormalized parameter encoding the one-loop functional-measure quantum gravity correction such that $10^{-2}$ ${\mathrm{GeV}}^{4}≲\gamma ≲10^{-1}$ ${\mathrm{GeV}}^{4}$. In this way, experimental data seem to favor the presence of a non-trivial functional measure. This suggests that a high-temperature scenario might be used to test the functional-measure correction and ultimately serve as an experimental probe for quantum gravity.

## Data Availability

The datasets generated during and/or analyzed during the current study are available from the corresponding author upon reasonable request.
